# Supplementary material for: Polymorphisms Analysis of BMP15, GDF9 and BMPR1B in Tibetan Cashmere Goat (Capra hircus)
Source: Genes (Basel). 2023 May 18;14(5):1102. doi: 10.3390/genes14051102 (PMC10218189; doi:10.3390/genes14051102)
Supplement: Supplementary file 1 [file genes-14-01102-s001.zip › genes-2360132-supplementary.pdf]

Supplementary Table and Figures

Supplementary Table S1. Positions of mutations with changes in bases and its effect with amino acids in BMP 15 of Tibetan cashmere goat

Mutation	Position(bp)	Base change	Codon	Amino acid change
G732A	732	G-A	AAG*-AAA	Lysine (K) unchanged
C805G	805	C-G	C*AA-GAA	Glutamine (Q) - Glutamic acid (E)

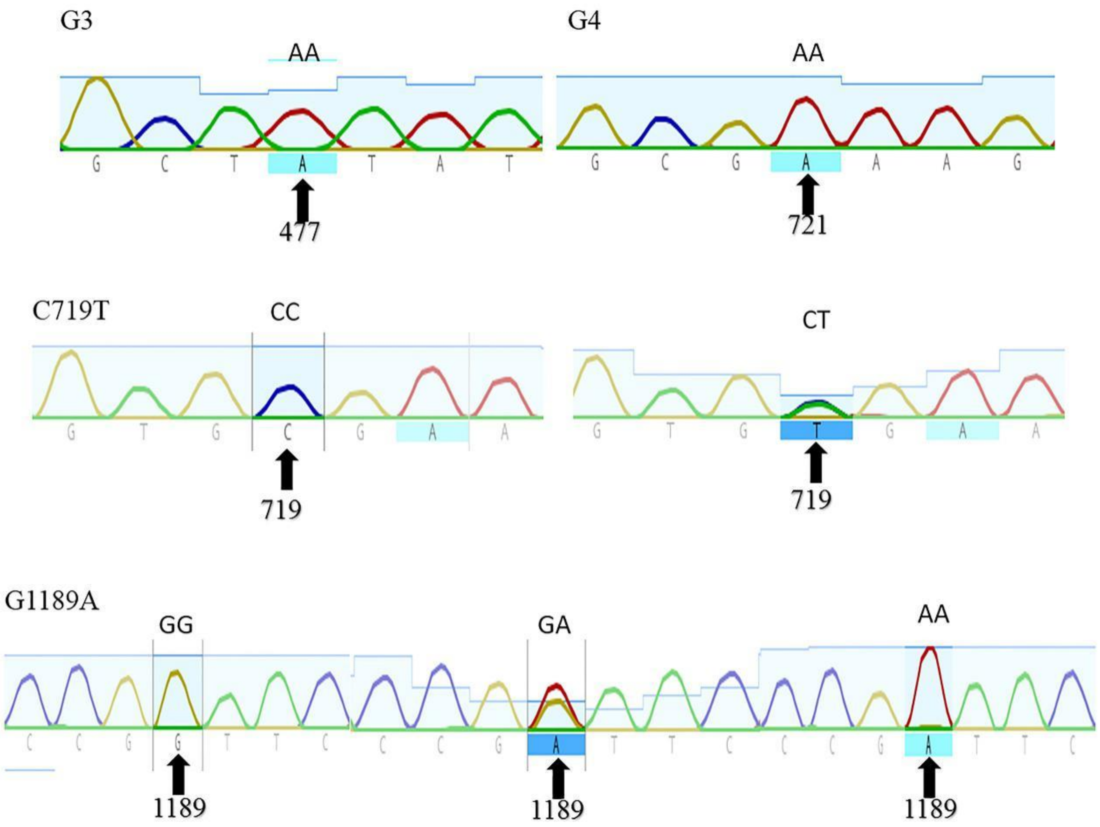

Supplementary Figure S1. Sequence maps of GDF9.

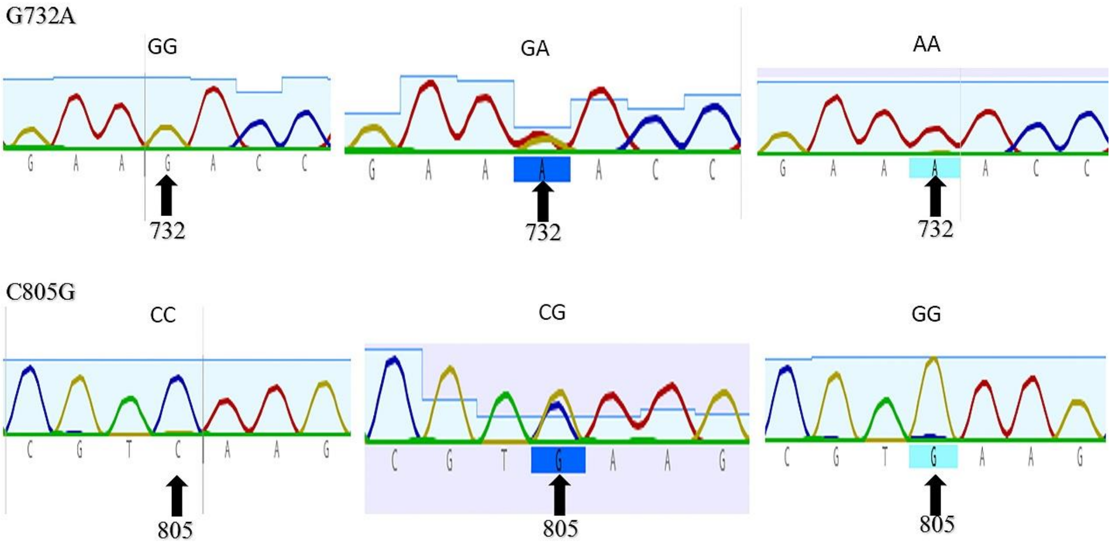

Supplementary Figure S2. Sequence maps of *BMP15*.
